# Montreal Battery of Evaluation of *Amusia*: Validity evidence and norms for adolescents in Belo Horizonte, Minas Gerais, Brazil

**DOI:** 10.1590/S1980-57642012DN06040008

**Published:** 2012

**Authors:** Marília Nunes-Silva, Vitor Geraldi Haase

**Affiliations:** 1 Doctoral student in the Neuroscience Program at the Universidade Federal de Minas Gerais (UFMG), Belo Horizonte MG, Brazil. Master’s degree in developmental Psychology at UFMG. Graduated in Psychology at UFMG and in Music at Universidade do Estado de Minas Gerais (UEMG).; 2 Full Professor in the Psychology Department at UFMG. Graduated in Medicine at the Universidade Federal do Rio Grande do Sul (UFRGS), Porto Alegre RS, Brazil. Master’s Degree in Applied Linguistics at the Pontifícia Universidade Católica do Rio Grande do Sul (PUCRS), Porto Algre RS, Brazil. Ph.D. in Medical Psychology at the Ludwig-Maximilians-Universität in München.

**Keywords:** music, cognition, neuropsychological tests, validation studies, Montreal battery

## Abstract

The Montreal Battery of Evaluation of Amusia (MBEA) is a battery of tests that assesses six music processing components: scale, contour, interval, rhythm, metric, and music memory. The present study sought to verify the psychometric characteristics of the MBEA in a sample of 150 adolescents aged 14-18 years in the city of Belo Horizonte, Minas Gerais, Brazil, and to develop specific norms for this population. We used statistical procedures that explored the dimensional structure of the MBEA and its items, evaluating their adequacy from empirical data, verifying their reliability, and providing evidence of validity. The results for the difficult levels for each test indicated a trend toward higher scores, corroborating previous studies. From the analysis of the criterion groups, almost all of the items were considered discriminatory. The global score of the MBEA was shown to be valid and reliable (*r*_K-R20_=0.896) for assessing the musical ability of normal teenagers. Based on the analysis of the items, we proposed a short version of the MBEA. Further studies with larger samples and amusic individuals are necessary to provide evidence of the validity of the MBEA in the Brazilian milieu. The present study brings to the Brazilian context a tool for diagnosing deficits in musical skills and will serve as a basis for comparisons with single case studies and studies of populations with specific neuropsychological syndromes.

## Abstract (pt)

A Montreal Battery of Evaluation of Amusia (MBEA) é uma bateria de testes que avalia funções musicais referentes a seis componentes do processamento musical: escala, contorno, intervalo, ritmo, métrica e memória musical. O presente estudo objetivou verificar as características psicométricas da MBEA em uma amostra de 150 adolescentes de 14 a 18 anos da cidade de Belo Horizonte e compor normas específicas para essa população. Foram utilizados procedimentos estatísticos que permitiram explorar a estrutura dimensional da MBEA e dos itens componentes, avaliar a adequação dos itens a partir de dados empíricos, verificar a confiabilidade e levantar evidências de validade a partir da análise fatorial. A análise de grupos-critério indicou que quase todos os itens podem ser considerados discriminativos. O índice global da MBEA se mostrou válido e fidedigno (*r*_K-R20_=0,896) para avaliar as habilidade musicais de adolescentes normais. A partir da análise dos itens foi proposta uma versão reduzida da MBEA. O presente estudo, além de trazer para o contexto brasileiro um instrumento para o diagnóstico de déficits de funções musicais, poderá servir como base de comparação para estudos de caso simples e estudos posteriores em populações com síndromes neuropsicológicas específicas.

## INTRODUCTION

Music is a complex cognitive ability that requires efficient brain mechanisms to be processed. Failure of these mechanisms can result in different types of clinical musical deficits. Neurologists have analyzed disorders of musical functioning in patients with brain illness since the latter half of the 20^th^ century in an attempt to associate brain lesions with specific brain deficits. Deficits in musical processing are grouped under the term *amusia*, which was first introduced by the German doctor and anatomist August Knoblauch in 1888 to describe a specific disorder that results from lesions to the motor center for tones.^1^

The term *amusia* is still controversial, with no consensus on the classification of the many forms and definitions of this syndrome, despite many studies of these musical deficits.^2^ Amusia has also been described under the terms *note deafness, tone deafness, tune deafness*, and *dysmelodia*.^3^ Many classifications have been proposed for the different kinds of amusias. According to Johnson and Graziano (2003),^1^ for example, Knoblauch proposed a detailed cognitive model for music processing, suggesting nine different types of amusias from clinical observations of patients. Benton (1977)^4^ also classified musical deficits based on clinical observations, observing that amusias could manifest in several ways. Benton (1977)^4^ classified amusias as receptive amusia, musical alexia, musical amnesia, rhythm disorders, vocal or oral-expressive amusia, instrumental amnesia or musical apraxia, and music agraphia. Marin and Perry (1999)^5^ defined amusias as acquired clinical disorders attributable to brain damage in the fields of reading, writing, and musical perception and performance and proposed a classification of amusias according to a hierarchical order of processing. The authors considered the existence of specifically perceptual amusias, amusias that involve symbolic systems of reading and writing (based on previous knowledge), and other amusias related to vocal performance or motor activities. Levitin (1999)^2^ also proposed a taxonomic system for classifying the various forms of amusias (i.e. tone-deafness), grouping them according to four different deficits: production deficits, perceptual deficits, memory deficits, and symbolic manipulation deficits (either music reading or writing). All of these classifications consider amusias as a complex and heterogeneous group of disorders of music processing that affect either one or more components of musical cognitive processing. Therefore, amusias can affect the performance and perception of melodies or their components (pitch, loudness, timbre, duration, and harmony) as well as symbolic systems of musical reading and writing.

Amusias can be categorized into two types: acquired amusias, resulting from disease or brain damage caused by accidental injury, and congenital amusias that are present since birth and may be due to hereditary factors.^3,6^ Congenital amusia has been systematically investigated only recently.^7^ Hyde and Peretz (2004)^6^ defined congenital amusia as a lifelong inability to process musical skills, despite normal intelligence, memory, and language. Individuals with congenital amusia do not develop basic musical abilities, presenting deficits in tonal processing, exhibiting difficulty recognizing familiar sounds, distinguishing one tune from another, and singing tunes or producing rhythmic patterns.

**Figure 1:**
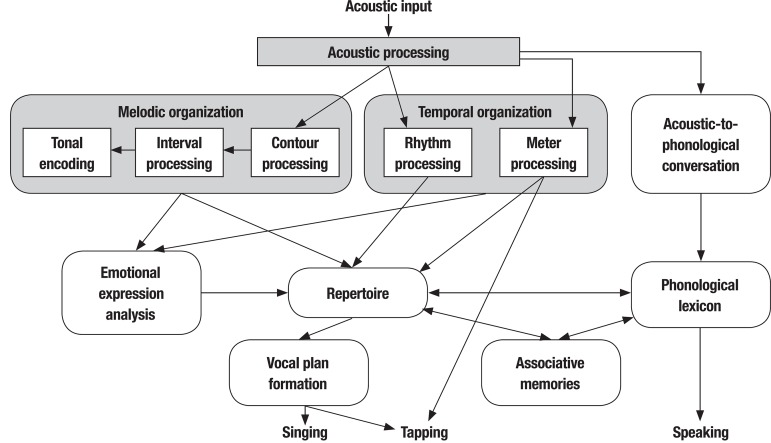
Cognitive-neuropsychological model of music processing (Adapted by permission from Macmillan Publishers Ltd: [Nature Neuroscience] (Peretz, I. & Coltheart, M. Modularity of music processing, Vol 6 (7), 688–691). Copyright (2003).

According to Peretz, Champod, and Hyde (2003),^8^ musical abilities may be compromised in a very selective way in both acquired and congenital amusias. Brain damage or deficits may interfere with musical function, whereas other domains, such as intelligence and language, remain intact. Moreover, not all musical abilities are equally affected. The processing of music relies on a complex and specific cognitive structure based on the modular organization of music in the brain. According to Peretz and Coltheart (2003),^9^ musical functions are part of a distinct mental module with its own system of information processing and specific neural substrates. This module consists of processing subsystems, whose domains are restricted to particular aspects of music. Thus, neurological abnormalities can either damage one or more of the processing components or interfere with the passage of information between components. This perspective and studies of individuals who suffer selective deficits in musical abilities because of brain injuries, allowed the development of models to understand the components involved in the processing of music perception, such as the model described by Peretz and Coltheart (2003).^9^ This neuropsychological model of musical cognitive processing, which specifies the components involved in perception and musical memory and their possible interactions, is depicted in Figure 1.

In this model, the auditory input has some aspects that elicit the action of the language processing system and other aspects that trigger the musical processing system. The lyric component of song is processed in the language processing system in parallel with the musical processing system.^9^ The musical auditory input is analyzed by two independent and parallel systems with specific functions: one for the melodic dimension (related to variations in sound frequency), represented by the contour (direction of pitch sequences in a melody), scale (related to tonal functions), and interval (range size between two different pitches), and one for the temporal dimension (related to variations in the duration of sounds), represented by the rhythm (grouping of events according to temporal proximity) and metric organization (basic temporal regularity or pulse).

The outputs of melodic and temporal dimensions are sent to the *repertoire*, which is conceived as a perceptual representation system that contains all of the representations of musical phrases to which the subject was exposed throughout their life. In this model, the emotional component refers to affective information provided by musical input and depends on two structures: the mode (i.e., the character of a scale that varies with the position of tones and semitones and their relationship to the tonic) and the tempo (i.e., the speed or pace of a piece).^8,9^

The neuropsychological cognitive model of musical processing was constructed from double dissociations observed in different studies of amusic individuals following brain injury. In acoustic processing, these studies allowed the differentiation of separate modules for processing music, language, and other environmental sounds.^5,10-14^ With regard to music perception, these studies support the existence of two dissociated and parallel routes for musical input: temporal and melodic.^13,15-18^ The melodic route is divided into three distinct modules: tonal encoding, contour, and interval.^15,16^ The temporal dimension has two distinct modules: rhythm and meter.^16.19^ According to Peretz et al. (2003),^8^ this model led to the development of the Montreal Battery of Evaluation of Amusia (MBEA), providing theoretical support for the battery as a tool for neuropsychological assessment.

The MBEA is a battery of tests that assesses musical abilities that has been developed and revised since 1987.^8^ The MBEA allows the diagnosis of different types of amusia by assessing musical abilities related to six components of musical processing presented in the neuropsychological model of musical cognitive processing, namely: Contour, Scale, Interval, Rhythm, Meter, and Musical Memory. The Contour test assesses the perception of a global form of a melody created from sequences of pitch direction (ascendant and descendant) of the melody. The Scale test assesses the tonal encoding of a melody that is related to tonal functions and harmonic structures. The Interval test evaluates the perception of distances between two successive pitches and is related to the analytical processing of the melodic domain. The Rhythm test assesses the perception of the grouping of events related to the temporal dimension of a melody with regard to the temporal proximity of consecutive sounds without considering its periodicity. The Meter test evaluates the global perception of the temporal music domain with regard to the temporal regularity or pulse of a melody. The Memory test assesses the recognition of musical phrases after implicit storage.^8^

The MBEA has been used in studies of populations of individuals with brain injuries with different etiologies to assess various types of amusia and was shown to be useful for this purpose.^10,15,16^ Studies have used the MBEA to validate the battery.^7,8,20,21^ Satisfactory results were obtained from a psychometric perspective. Peretz et al. (2003)^8^ estimated that, although the data obtained for each test were asymmetric (i.e., tending toward higher scores), the overall index (i.e., the average scores on the six tasks of the MBEA) followed a normal distribution and thus constituted a good index of perception and musical memory that can be used to distinguish between normal and deficient performance in the general population.

Peretz et al. (2003)^8^ reported that the concurrent validity of the MBEA was derived from Gordon's Musical Aptitude Profile tests. The study included a group of 68 firemen in training who obtained similar and positively correlated scores (r=0.53, p< 0.001) on both tests. According to Peretz et al.,^8^ the MBEA also has test-retest reliability (r=0.75, p<0.01). With regard to the diagnostic value of the MBEA for detecting amusia in the general population, Peretz et al.^8^ conducted a study to determine whether 27 healthy individuals who declared themselves amusical truly had a deficit in their skills of musical perception. The results showed that, as a group, their performance was lower than the control group for each MBEA test, thereby confirming their subjective experience. This outcome indicates that the MBEA can serve as a useful tool for diagnosing amusia not only in patients with brain injuries but also in the general population.

In Brazil, research in music and cognitive neuropsychology is incipient, with a lack of studies on deficits in musical processing. Nevertheless, some research efforts have been undertaken,^22-26^ mainly in musical education. Despite these efforts, we found no validated instruments in the Literatura Latino Americana em Ciências da Saúde (LILACS) or Scientific Electronic Library Online (SciELO) databases, evaluating musical deficits in the Brazilian context. The diagnosis of amusia is reached based on clinical observations of patients with brain damage, with no specific criteria to distinguish neurological conditions of musical deficits from other causes of musical deficiencies in the musical education context, especially with regard to cases of congenital amusia.

Studies conducted by the authors of the present work^27^ to adapt the MBEA for use in the Brazilian context permitted verification of the relevance of its items and adequacy of its constructs to allow its use in adolescents in the city of Belo Horizonte. The evaluation of the relevance and adequacy of means, and the layout of the questions and instructions in the test setting, mode of application, and method of categorization were also satisfactory for the use of the MBEA in the Brazilian context. Following this first study, the present investigation sought to assess the psychometric characteristics of the MBEA and develop norms for the adapted version of the MBEA^27^ based on a sample of Brazilian adolescents from the city of Belo Horizonte.

## METHODS

**Participants.** The psychometric parameters of the MBEA were investigated in a convenience sample of 150 individuals who had no formal musical education, aged between 14 and 18 years. The sample was stratified according to 1-year age groups. In each age stratum, 30 individuals were equally divided between both sexes. The participants were secondary school students in Belo Horizonte, Minas Gerais, Brazil. The sample was also equally subdivided by type of educational institution (i.e., state-run, city-run, and private).

**Materials.** Adapted version of the Montreal Battery of Evaluation of Amusia (MBEA): The MBEA was adapted for use with adolescents aged 14 to 18 years in Belo Horizonte after a study that examined the adequacy of its constructs, items, and application procedures.^27^ The MBEA assesses six components of music processing: Contour, Interval, Scale, Rhythm, Meter, and Musical Memory. The MBEA stimuli consist of 30 original musical phrases for each test, which were composed according to the Western tonal system comprising a total of 180 items. For the evaluation of Contour, the items are identical melodies presented in pairs. Half of the items have one note altered in the second melody according to the direction of pitch (ascendant to descendant and vice-versa), while the other half of the pairs remains unchanged. The interval and scale of the melodies remains unaltered. Modified and non-modified phrases are pseudorandomly dispersed among a total of 30 items. The subject's task is to identify whether one of the phrases is modified or not. The Interval test is similar to the Contour test, but the note is altered in the modified items according to the extent of the pitch in relation to a previous note (in terms of semi-tone distance), keeping the original scale and contour. In the Scale test, the manipulations of the modified items consist of modifying the pitch to be out of scale, maintaining the original melodic contour. In the Rhythm test, groupings by temporal proximity are manipulated by changing the durations of two adjacent tones while the same meter and total number of sounds were maintained. For these first four tests, an additional item, the catch trial, consists of strategic trials that had to be answered correctly for responses to be considered. This item contains pairs of melodies that are clearly different to determine whether the individual remains attentive throughout the test session. In the Meter test, half of the 30 phrases were composed in a duple meter, and the other half were composed of a triple meter. The subjects are required to categorize the melodies as a waltz or march. Finally, in the Memory Recognition test, the participants are required to recognize 15 of the previously presented phrases pseudorandomly interspersed with 15 novel melodies. The MBEA is individually applied, and has a duration of approximately 90 min.^8,27^ Examples of the musical stimuli and test construction are outlined in detail in Peretz et al. (2003).^8^

**Procedures.** The project was approved by the review board of the Federal University of Minas Gerais (ETIC no. 318/08). After obtaining permission from the school principals, the research project was presented in the classrooms. The parents or guardians of the interested students received an invitation letter and provided informed consent. The inclusion criterion was absence of formal musical education. All 150 participants were individually subjected to the MBEA in adequate and properly prepared rooms provided by the schools. Testing was conducted by a team of undergraduate psychology students with training in psychometrics, which was led by the first author of this article.

**Statistical analyses.** Item dimensionality and homogeneity were analyzed using exploratory factor analysis (EFA). Item difficulty was estimated by percent accuracy (i.e., the number of individuals who correctly answered an item divided by the total number of participants who responded to the item), with higher difficulty indices indicating easier items. Discrimination indices were calculated based on criterion groups in the higher and lower quartiles using both the *D* index and *t*-test. The internal consistency of the items was assessed using the Kuder-Richardson (K-R20) formula. Norms for statistical analyses of single case studies were built, estimating mean scores and standard deviations for each gender and age stratum according to the method proposed by Crawford and Howell (1998).^28^ Percentile norms were also estimated because this scale directly expresses the rarity of scores.

## RESULTS

**Item dimensionality.** According to the pre-specified MBEA model, each domain should be unidimensional.^8,9^ The EFA conducted for the 30 items in each of the six MBEA components using the principal component analysis method revealed that only the results for the Meter test were adequate according to the Kaiser-Meyer-Olkin test (KMO=0.659). Twenty-six of the 30 Meter items loaded on the same factor but explained only 15.95% of its variance.

**Item difficulty.** The difficulty indices for the several MBEA domains varied between 44.7% and 100%, with 84.44% above 70%. Item 1 from Recognition Memory was the only item with a difficulty index of 100%, indicating that it was extremely easy.

**Item discrimination.** Criterion groups were established according to performance. Individuals with performance above the 73^rd^ percentile were allocated to the high performance group. The group of participants whose performance was below the 27^th^ percentile was designated as the low performance group. The D index results for each test are shown in Table 1.

**Table 1:** D indexes for MBEA Tests.

Items	Scale	Contour	Interval	Rhythm	Meter	Memory
Item 1	18.2	43.0	21.7	17.0	42.6	0.0
Item 2	44.0	23.7	39.3	12.0	31.3	3.7
Item 3	28.0	14.7	21.4	44.0	31.0	6.0
Item 4	20.6	20.7	16.6	39.2	27.2	3.7
Item 5	6.7*	31.3	20.8	6.7	5.6	7.4
Item 6	21.2	31.0	34.6	6.5	40.4	55.9
Item 7	-----**	29.3	16.5	8.0	36.6	17.4
Item 8	42.5	27.7	21.2	34.7	34.7	31.3
Item 9	62.2	36.0	23.9	18.5	29.0	16.1
Item 10	15.4	30.7	28.3	16.0	38.9	1.9
Item 11	27.6	16.0	38.7	10.0	22.1	17.1
Item 12	28.4	36.3	14.3	26.2	23.8	17.4
Item 13	24.8	9.0	37.4	14.7	16.7	24.3
Item 14	32.6	16.3	32.2	------	9.0	7.8
Item 15	18.0	11.3	11.8	24.2	24.1	18.9
Item 16	30.5	11.0	29.3	18.0	50.0	14.3
Item 17	21.2	22.3	21.3	18.7	44.4	9.3
Item 18	1.4	38.7	16.8	29.2	38.6	11.1
Item 19	21.1	-0.7	23.2	17.7	24.1	28.6
Item 20	11.3	15.3	8.3	12.0	33.0	1.9
Item 21	15.4	-----	38.0	23.2	13.0	9.3
Item 22	20.8	12.3	24.8	18.7	25.8	37.4
Item 23	11.5	40.7	14.9	22.0	14.8	7.4
Item 24	23.1	21.7	35.2	6.0	20.4	16.7
Item 25	16.8	44.7	25.0	10.2	22.1	4.6
Item 26	34.4	35.0	39.4	20.0	22.2	18.8
Item 27	48.5	7.0	8.0	8.5	29.6	13.0
Item 28	64.1	27.3	------	30.2	34.7	13.4
Item 29	30.9	4.3	15.2	18.2	36.8	16.7
Item 30	29.8	5.3	49.8	20.2	22.2	5.6
Item 31	40.1	40.0	42.4	22.0		

* In bold, items not discriminative also by t Test (p>0.05);

** Catch Trials.

**Reliability.** The K-R20 coefficient for the entire sample of items was 0.896. Internal consistency was also high when considering each of the main component subgroups, with the exception of Recognition Memory. The K-R20 was estimated to be 0.848 for Melodic Organization, 0.775 for Temporal Organization, and 0.582 for Recognition Memory. Meter was the only isolated music component for which the K-R20 coefficient was higher than 0.70.

**Factor validity.** An EFA analysis was conducted using the average of raw scores over all of the subtests. The KMO test resulted in a value of 0.858, indicating the adequacy of the sample. The Bartlett test of sphericity yielded p<0.0001, indicating that the correlation matrix was different from the identity matrix.^29^ Using the principal component extraction method, observing one factor that could explain 56.58% of the variance was possible. The factor loadings are shown in Table 2.

**Table 2:** Principal component analysis of MBEA.

MBEA tests	Factor loading	h^2^
Scale	0.818	0.669
Contour	0.871	0.759
Interval	0.821	0.675
Rhythm	0.647	0.419
Meter	0.580	0.337
Musical Memory	0.732	0.536

h^2^: Communalities (proportion of variance that can be explained by the factor).

**Figure 2:**
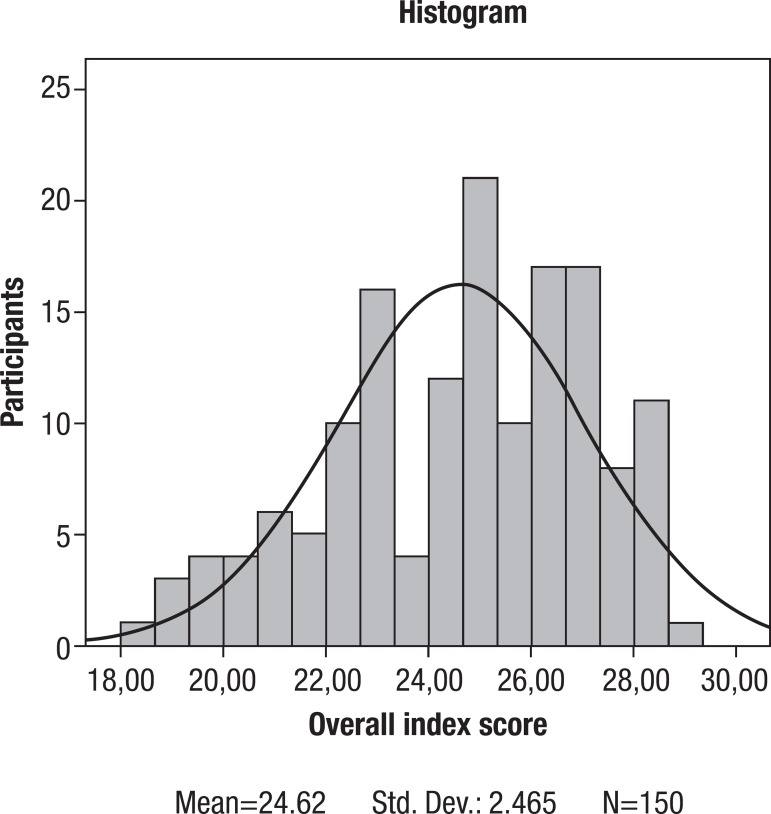
Distribution of global composite scores obtained on the MBEA for 150 normal adolescents. The mean corresponds to a score of 24.6 and the standard deviation to 2.5.

**Participant performance profile on the MBEA.** The data obtained from the adolescents indicated that the results on the temporal tests (Rhythm, M=26.3, SD=2.7; Meter, M=24.7, SD=4.1; Recognition Memory, M=26.8, SD=2.4) were greater than the results on the melodic tests (Scale, M=23.2, SD=3.6; Contour, M=23.9, SD=3.3; Interval, M=22.8, SD=3.7). Perfect scores were obtained for all of the tests, with the exception of the Contour test. However, no perfect score was obtained for the overall index (M=24.6, SD=2.5). Although the data for each test were skewed toward higher scores, the average score over the six tests used to generate the overall index followed a normal distribution (Figure 2).

Considering the overall index, at the lower extreme, we found that five individuals (approximately 3% of the sample) obtained scores that were two standard deviations less than the mean. These results are considered to indicate abnormal performance, and these individuals may likely be considered amusic.

**Establishment of norms for the MBEA.** To establish norms that can serve as basis for single case studies, the data given in Table 3 contain the means and standard deviations for each sex (male and female) for each age of the sample (14, 15, 16, 17, and 18 years).

**Table 3:** Norms of MBEA for single case studies (Belo Horizonte-MG).

Gender	Age	Scale			Contour			Interval			Rhythm			Meter			Memory			Average	
		**M**	**SD**		**M**	**SD**		**M**	**SD**		**M**	**SD**		**M**	**SD**		**M**	**SD**		**M**	**SD**
Female	14	23.67	3.48		23.40	3.46		23.53	3.04		24.87	4.21		25.13	2.90		26.87	2.26		24.58	2.48
	15	22.33	4.30		23.73	3.75		22.33	4.30		25.80	3.05		23.47	3.98		26.00	2.62		23.94	2.91
	16	21.93	2.60		23.53	2.33		21.80	2.98		27.07	2.28		25.40	3.11		27.07	2.40		24.47	1.43
	17	22.27	3.69		23.40	4.00		21.73	4.03		25.93	2.31		24.60	4.53		26.33	2.89		24.04	2.79
	18	23.20	3.34		23.73	3.33		23.13	3.72		26.73	1.75		24.73	3.79		26.93	2.05		24.74	2.19
Male	14	22.87	3.14		23.13	2.95		22.00	2.85		26.27	2.74		23.40	4.07		27.47	1.88		24.19	2.16
	15	24.73	2.94		23.60	3.68		23.53	3.50		25.73	2.58		25.93	3.75		26.80	2.31		25.06	2.32
	16	23.00	4.88		25.00	3.68		23.40	4.24		26.53	2.92		25.27	4.85		27.33	2.13		25.09	2.94
	17	24.13	3.09		24.73	3.10		23.87	3.98		27.20	2.27		25.27	3.45		26.73	2.25		25.32	2.32
	18	23.73	4.04		24.67	2.87		23.13	4.41		26.47	2.50		24.07	5.64		26.80	2.93		24.81	3.03

n=15 for each age and gender. M: mean; SD: standard deviation.

Preliminary norms for adolescents aged 14 to 18 years were also established by converting the raw scores into percentiles (Table 4).

**Table 4:** Norms of MBEA for adolescents aged 14 to 18 years (Belo Horizonte-MG).

Percentile	Scale	Contour	Interval	Rhythm	Meter	Musical memory	Average*
10	18.10	19.10	17.00	22.00	19.00	23.00	21.00
20	20.00	21.00	20.00	24.00	22.00	25.00	22.50
30	21.00	22.00	21.00	25.00	23.00	26.00	23.17
40	22.40	23.00	22.00	26.00	24.00	27.00	24.33
50	23.00	25.00	23.00	27.00	25.00	27.00	25.00
60	24.00	25.60	25.00	27.00	27.00	28.00	25.67
70	26.00	26.00	25.00	28.00	28.00	28.00	26.33
80	27.00	27.00	26.00	29.00	28.00	29.00	26.67
90	28.00	28.00	27.00	29.00	29.00	29.00	27.67

n=150.

* Average corresponds to the overall index over the six tests of battery.

**Proposal for a short version of the MBEA.** Based on the analysis of the items, we proposed a short version of the MBEA by considering:

[1] items with higher D indexes that could better discriminate the different levels of musical abilities in the general population,
[2] items with lower levels of difficulty to allow greater variability in the results,
[3] items with satisfactory factor loading, and
[4] items with adequate item-total correlation coefficients.

Importantly, the Musical Memory test depends on items from other tests because it requires the participants to recognize the melodies that they heard in previous tests. Moreover, the tunes should be equal for all of the tests. Therefore, to compose a short version of the MBEA, equal melodies were verified for all of the tests of the battery that showed the best psychometric results throughout the battery while maintaining the equal proportion between items with same and different answers (Table 5).

**Table 5:** Proposal for a short version of the MBEA.

Items	Scale	Contour	Interval	Rhythm	Meter	Memory
ex1	24/29*	20	18	3	catch trial	5/1
ex2	15	4	24/2	17	4	31
ex3	*	*	*	*	15	*
ex4	*	*	*	*	27	*
1	1^+^	5	19	6	5	5
2	2	25	24	21	12	32
3	3	9	11	16	28	19
4	4	21	22	22	22	33
5	5	26	23	15	10	34
6	6	11	21	24	29	35
7	catch trial	12	13	13	2	14
8	7	7	7	10	14	12
9	8	13	17	1	11	36
10	9	7	9	7	8	7
11	10	19	8	18	16	37
12	11	23	7	2	19	38
13	7	13	14	12	3	39
14	12	24	26	catch trial	24	9
15	13	17	18	26	9	40
16	7	3	3	3	20	16
17	14	5	12	5	26	41
18	15	6	20	14	7	10
19	16	15	16	19	30	42
20	17	22	15	23	1	13
21	18	catch trial	4	8	13	21
22	19	1	10	22	25	22
23	20	2	25	5	21	43
24	5	14	5	25	17	44
25	21	7	7	11	6	2
26	22	8	6	20	18	24
27	23	4	5	7	23	11
28	13	20	catch trial	9	15	45
29	24	18	1	4	27	46
30	25	16	2	13	4	8
31	26	10	13	17		

+ Numbers correspond to melodies composed for the tests. The items that will be kept are in grey.

* 24/29= melody 24, corresponding to item 29 of the same test; 24/2=melody 24, item 2; 5/1=melody 5, item 1.

The melodies composed for all of the tests were identified during the entire battery. We identified 26 main melodies present in all of the tests, with the exception of the Musical Memory test, for which 15 additional melodies were composed. For the short version, we excluded items with poor psychometric results and repeated items, leaving only 14 tunes comprising the entire battery. The identified melodies in the table chosen to compose the short version of the MBEA were 1, 2, 5, 6, 7, 8, 9, 12, 13, 19, 21, 22, 23, and 26. We suggest [1] replacing ex. 1 of the Scale test with melody 24, corresponding to item 29 of the same test, [2] replacing ex. 2 of the Interval test with melody 24, item 2, so that they are not the same as the test items, [3] replacing ex. 1 of the Memory test with melody 5, item 1, because tune 4 was excluded from the short version, and [4] retaining the catch trials because they determine whether the person remains alert during testing. Thus, in the short version, the first four tests (Scale, Contour, Interval, and Rhythm) have seven trials that contain pairs of identical melodies, seven trials that include a different comparison melody and one catch trial in random order. For the Meter test, half of the trials correspond to a binary structure (march), and half correspond to a ternary structure (waltz). Finally, in the Memory test, half of the trials correspond to a melody that was previously heard, and half of the trials correspond to a novel melody.

## DISCUSSION

The results of the psychometric quality analysis of the items indicated that the test was considered relatively easy, which is consistent with the findings of previous studies involving a Canadian sample.^8^ With regard to discrimination, although most of the items presented positive D indexes, they showed little discriminative value with regard to the criterion groups. The sample was composed of a non-clinical group, and the test itself was easy, which likely contributed to the low D indexes. Notably, however, the *t*-test revealed that most of the items could be considered significantly discriminative for this population.

The analysis of the items' dimensionality from EFA indicated that the items in each test could not be reduced to a single dimension or variable, with the exception of the metric test. Nonetheless, the items of the metric test were responsible for a small portion of the explained variance, indicating that the items were distributed in more than one factor. This result was expected because the sample was homogeneous, consisting of healthy individuals, and the test was very easy for this population, reflected by the distribution of the data. Although the distribution of the data can be considered normal for most tests, it shows a tendency toward negative skewness.

For this reason, a one-factor model of the MBEA observed from factor validity data would most likely be confirmed. The factor analysis for a one-factor model was performed with the total scores of the battery, which showed greater variability in the sample. Moreover, as this was a study focused on a non-clinical sample, the tendency would be to find more general results as obtained in previous studies. Therefore, the obtained factorial matrix was similar to the theoretical factor concerning the musical perception global ability, indicating that the overall index of the MBEA is appropriate to measure these abilities in the adolescent population in Belo Horizonte.

The coefficient of precision was high (*r*_K-R20_=0.896) considering all items of the battery. This result indicates adequate reliability with regard to the whole instrument for assessing musical ability and corroborates the results obtained from the EFA, indicating that the MBEA is a good instrument for assessing musical ability in the adolescent population in Belo Horizonte.

The findings of the present study are consistent with previous studies,^8^ provide an empirical basis for the model of music processing developed by Isabelle Peretz,^8,9^ and contribute to a better understanding of musical processing. However, some limitations should be highlighted, such as the sample size and its homogeneity, which resulted in the low variability of results on each specific test. The validity analysis did not include any other instrument adapted for Brazil to assess the constructs because no such instrument was available. Using other strategies in future studies may provide further evidence for the validity of the MBEA. Nevertheless, the lack of studies demonstrates the importance of the present study because an instrument that assesses musical ability deficits in the Brazilian context is needed.

The validation of the MBEA for the assessment of amusia, both congenital and acquired, in a Brazilian sample may allow a more accurate diagnosis of musical ability deficits and help estimate the impact of clinical interventions based on elements of music. We may then be able to identify preserved and compromised domains of musical processing in individuals on the neuropsychological domain and consequently develop more effective rehabilitation strategies. The present study brings to the Brazilian context a tool for diagnosing musical ability deficits, serves as a basis for single case study comparisons and future studies in populations with specific neuropsychological syndromes, and may contribute to future music and cognition research in Brazil. In the musical education context, the MBEA may be able to distinguish between neurological conditions and others causes of musical deficiencies,^22^ especially in cases of congenital amusia. Notably, the MBEA evaluates musical perception and does not assess musical performance skills, such as singing and playing. The MBEA also does not include all musical perception abilities. Evaluations of the emotional component of the melodies and perception of harmony, for example, must be performed using additional batteries.^30^

The item analysis allowed the selection of items to compose a short version of the MBEA based on their psychometric properties. Some barriers were found with regard to using the same melodies in different tests and the difficulty maintaining the proportion of items with equal and different answers. Nevertheless, we were able to exclude at least five of the worst items in each test and generate a version with 14 melodies that had satisfactory psychometric results throughout the battery without repetition. Administering the MBEA required approximately 90 min for each individual. The test requires sustained attention and can be very tiring for the participant. The use of a short version of the MBEA in validation studies may yield better psychometric results and enable quicker evaluation of musical abilities.

The present study provides evidence of the validity and reliability of the MBEA for the target population and demonstrates that the overall index of the MBEA is appropriate for assessing musical abilities in normal adolescents in Belo Horizonte. Future studies should provide additional psychometric data and include clinical populations with specific deficits.
